# Editorial: Advanced cell culture technologies to boost cell-based therapies, volume II

**DOI:** 10.3389/fbioe.2022.999765

**Published:** 2022-08-30

**Authors:** Dominik Egger, Jan Hansmann, Cornelia Kasper, Dimitrios Kouroupis

**Affiliations:** ^1^ Institute of Cell and Tissue Culture Technologies, Department of Biotechnology, University of Natural Resources and Life Sciences, Vienna, Austria; ^2^ Department for Tissue Engineering and Regenerative Medicine, University Hospital Würzburg, Würzburg, Germany; ^3^ Translational Center Regenerative Therapies (TLC-RT), Fraunhofer Institute for Silicate Research ISC, Würzburg, Germany; ^4^ Department of Orthopedics, Division of Sports Medicine, University of Miami, Miller School of Medicine, Miami, FL, United States; ^5^ Diabetes Research Institute, Cell Transplant Center, University of Miami, Miller School of Medicine, Miami, FL, United States

**Keywords:** 3D cell culture, physiological conditions, cell-based therapy, therapeutic potential, stem cells

Approved cells and cellular products (i.e., extracellular vesicles) for cell-based therapy applications carry a huge promise for the treatment of a broad variety of diseases and several (stem) cell therapies. However, the therapeutic potential of cells is not fully exploited at present. On the one hand, outdated culture conditions are still used during *in vitro* cultivation. On the other hand, technological hurdles block the way to efficient and safe cell-based therapy products. Traditional cell culture conditions do not represent the physiological conditions of the cellular native environment (Yamada et al., 2022). Conventional extensive 2D *in vitro* mesenchymal stem cells (MSCs) expansion, aimed at obtaining clinically relevant therapeutic cell numbers, results in detrimental effects on both cellular characteristics (e.g., phenotypic changes and senescence) and functions (e.g., differentiation capacity and immunomodulatory effects) (Kouroupis et al., 2017; Almeria et al., 2019; Egger et al., 2021; Kouroupis and Correa, 2021). Feng et al. presented an innovative cocktail of three small-molecule compounds, ACY (A-83–01, CHIR99021, and Y-27632), to increase MSCs propagation *in vitro*. ACY can promote MSC proliferation, inhibit senescence, and maintain cell phenotype (including genomic stability, stem cell potential, and homogeneity). Importantly, authors demonstrated that ACY can prevent the upregulation of HLA-DR and promote the secretion of immunomodulatory cytokines and chemokines, demonstrating its potential for clinical application.

The manufacturing of T cells is an important process for adoptive T cell immunotherapies. However, the effect of culture conditions during transduction and expansion on their migration, intra-tissue differentiation, metabolism, signal transduction or circulation behavior is largely unknown. Sudarsanam et al. gave an overview on current T cell expansion strategies and summarize the benefits and drawbacks of chimeric antigen receptor (CAR) T cell therapies observed in clinical trials. Based on these observations they formulate open questions that the scientific community should address to advance in this field. T cells are also an interesting tool for the adoptive immunotherapy of SARS-CoV-2 patients. In this regards, Bonifacius et al. demonstrate the feasibility of clinical-grade SARS-CoV-2-specific T cell manufacturing for adoptive T cell transfer in COVID-19. The magnetically enriched antiviral T cells remained functionally and phenotypically stable. With this approach it might be possible to support the immune system of COVID-19 patients which are at risk for sever disease.

It is now apparent that the mechanical properties of scaffold materials or the cell microenvironment and related mechanotransduction are of utmost importance in tissue engineering (TE) field. Specifically, in skeletal muscle tissue engineering (SMTE) structural and mechanical cues in the respective scaffold biomaterial, such as micro-architecture and stiffness, guide and induce muscle differentiation and are pivotal for the myogenic outcome. On this basis, Tomasch et al. shed light on the impact of culture conditions in fibrin hydrogels on a well-established murine myoblast cell line, C2C12, and the human C25 myoblast cell line. According to their findings, the effects of fibrin hydrogel elastic modulus on myoblast proliferation changed depending on culture type. Three-dimensional cultures impair specifically human myoblasts proliferation and differentiation compared to 2D cultures. Therefore, the results highlight the need to adapt parameters of 3D culture setups established for murine cells when applied to human cells.

Altering physical, chemical and biological parameters to support the therapeutic properties of cells is often accompanied by technological advances. Microphysiological/organ-on-a-chip systems aim to recapitulate the behavior of different cells, organs and even multiple-organs, and thus mimic complex physiological or pathological processes. Importantly, organ-on-a-chip systems can be considered a potent technology for drug discovery and an alternative to animal testing which in some cases raise ethical concerns from organizations such as People for the Ethical Treatment of Animals (PETA). Up-to-date, organ-on-a-chip technology has been used to control both intra- and inter-cellular communication processes as well as providing necessary architectural features seen in heart, lung, liver, kidney, skin, eye, and musculoskeletal tissues. However, major challenges still remain associated with the establishment of defined two- and three-dimensional spatially-resolved individual cell layers needed to form complex heterogenous architectural features of human tissue. Rothbauer et al. highlighted in their review article the importance of additive manufacturing techniques for organ-on-a-chip applications that may overcome existing challenges, such as inkjet printing, extrusion bioprinting, laser-assisted printing as well as stereolithography and two-photon polymerization techniques. Authors evaluated extrusion-based bioprinting as method of choice for organ-on-a-chip systems due to the broad availability of affordable bioprinters and usage of well-established hydrogel systems such as gelatin, collagen, alginic acid (alginate) or fibrin. Of note, extrusion-based printers can be easily built in a “do it yourself” manner using syringes and automated stages, whereas comparative evaluation of various bioprinting methods for organ-on-a-chip applications indicate that extrusion bioprinting is not toxic for the cells. Here, the authors point out that the organ-on-a-chip technology and bioprinting are not competing technologies but can be actually synergistic. Thus, the combination of technologies may enable the highest degree of environmental control for the generation of 3D tissue- and disease models. In a second review article, Rothbauer et al. summarized 1) the most significant recent developments in the field of microsystem approaches for osteoarthritis (OA) modeling, as well as 2) an OA-pathophysiology-based bioengineering roadmap. The authors point out that novel trans- and cross-disciplinary approaches are required to achieve the next generation of chip-based OA models with improved relevance and predictability.

In summary, this article Research Topic provides a comprehensive overview of the state of the art in advanced cell culture technologies to boost cell-based therapies, which we think will be of significant interest to the journal readership.
